# Research and innovation paving the way for climate neutrality in urban transport: Analysis of 362 cities on their journey to zero emissions

**DOI:** 10.1016/j.tranpol.2024.01.008

**Published:** 2024-03

**Authors:** Panayotis Christidis, Giulia Ulpiani, Marcin Stepniak, Nadja Vetters

**Affiliations:** a European Commission, Joint Research Centre (JRC), Seville, Spain; b European Commission, Joint Research Centre (JRC), Ispra, Italy; c European Commission, Joint Research Centre (JRC), Brussels, Belgium

**Keywords:** Urban transport, Climate neutrality, Research, Innovation, Smart cities

## Abstract

The EU Mission on Climate Neutral and Smart Cities is an ambitious initiative aiming to involve a wide range of stakeholders and deliver 100 climate-neutral and smart cities by 2030. We analysed the information submitted in the expressions of interest by 362 candidate cities. The majority of the cities’ strategies for climate neutrality include urban transport as a main sector and combine the introduction of new technologies with the promotion of public transport and active mobility. We combined the information from the EU Mission candidate cities with data from the CORDIS and TRIMIS databases, and applied a clustering algorithm to measure proximity to foci of H2020 funding. Our results suggest that preparedness for the EU Mission is correlated with research and innovation activities on transport and mobility. Horizon 2020 activities specific to transport and mobility significantly increased the likelihood of a city to be a candidate. Among the various transport technology research pathways, smart mobility appears to have a major role in the development of solutions for climate neutrality.

## Highlights

• Analysis of 362 candidate cities to the EU Mission on Climate Neutral and Smart Cities.
• Urban transport is a main sector for climate neutrality.
• Strategies combine new technologies, public transport and active mobility.
• Horizon 2020 activities significantly increased the likelihood of a city to be a candidate.
• Smart mobility has a major role in the development of solutions for climate neutrality.

## 1 Introduction

The EU Mission on Climate Neutral and Smart Cities is an ambitious initiative aiming to deliver 100 climate-neutral and smart cities by 2030. These cities will act as experimentation and innovation hubs to enable all European cities to follow suit by 2050. The EU Mission is a catalyser for new ways of collaboration and coordination that recognizes the importance and complexity of the challenge of climate neutrality in transport. The report “Mission-Oriented Research & Innovation in the European Union” (Mazzucato, 2018) identified mission-oriented policy as the key instrument to reframe Europe's approach to tackling grand societal challenges and to make them more practical and systemic so that Research and Innovation (R&I) investments can help attain specific, targeted and concrete goals.

The central feature of the EU Mission is the implementation of Climate City Contracts by a group of about 100 cities, selected on the basis of diverse criteria including the ambition, preparedness and impact demonstrated in their expression of interest. The contracts will set out plans for the cities to achieve climate neutrality by 2030 and will include an investment plan. While not legally binding, these contracts will constitute a clear and highly visible political commitment to the EU, national and regional authorities and citizens.

Cities are considered as key players in designing and implementing climate policy (Fuhr et al., 2018; Li et al., 2018; Kern, 2019). Several networks and initiatives at city level already demonstrate a serious commitment towards combating climate change, such as the Covenant of Mayors (Kona et al., 2018; Melica et al., 2018; Palermo et al., 2020), the C40 Cities Climate Leadership Group (Araos et al., 2016), and the International Council for Local Environmental Initiatives (Heikkinen et al., 2020). However, currently there is no definitive agreement on how climate neutrality targets are implemented, while heterogeneity in the definition of neutrality can lead to very different climate ambitions and actions. Since “two net-zero commitments can be dramatically different, aiming for different timelines, covering different kinds of greenhouse gas (GHG) emissions, and relying on offsets to varying extents” (Fankhauser et al., 2022; NewClimate Institute & Data-Driven EnviroLab, 2020), a wide definition of gases, scopes and sectors is used in the Mission definition, allowing transparency in the overall process. Achieving climate neutrality will require a Mission City to reduce the GHG emissions from all sectors and sources within the city's boundary (as well as out-of-boundary emissions in the waste sector) to net zero by 2030 (European Commission, 2021).

From the technology point of view, several options for the reduction of GHG emissions are available for wide-scale implementation. Shifting fuel consumption from conventional technologies to carbon-free or carbon-neutral options would result in direct emission reductions. Examples include electric cars, hydrogen and fuel cells or synthetic fuels, as long as the energy required for the production of electricity or alternative fuels is itself of low carbon content. For public transport, a transition to fully carbon-neutral busses and urban trams and trains and an increase of the quality of service could provide an alternative to passenger cars, while contributing to the reduction of GHG emissions. Legislative measures, such as the constitution of Zero Emission Zones can drastically limit the access of internal combustion vehicles to specific urban areas where vulnerabilities are most critical. The promotion of public transport, cycling and walking can accelerate modal shift to cleaner modes, with additional benefits in terms of health, congestion, accidents and noise (Bernardino et al., 2015). Sustainable Urban Mobility Plans (SUMPs) have been developed in a large number of cities and have already demonstrated a major contribution towards the reduction of the negative impacts of transport at urban level (Pisoni et al., 2019). For urban freight, green last mile delivery options are available, including distribution by electric vans or cargo bikes (Giordano and Christidis, forthcoming). New technologies and business models such as telework, online shopping or e-health can decrease the total number of trips and bring significant savings in terms of emissions (Lopez Soler et al., 2021). In a similar fashion, emerging solutions in the fields of micro-mobility and Mobility as a Service (MaaS) can –under certain conditions-reduce the carbon footprint of mobility and stimulate innovation (Pisoni et al., 2022).

There is a wide range of urban and transport planning options that can lead to more carbon-neutral, liveable and healthier cities (Nieuwenhuijsen, 2020). Nevertheless, each city has its own priorities and challenges which require a combination of multiple innovative measures (Shang and Lv, 2023). In most cases, the adoption of new measures for urban transport and mobility requires a certain degree of experimentation and innovation, on both technological (Li et al., 2022) and institutional aspects (Nilssen and Hanssen, 2022). An extensive survey of pilot applications at city level (Wimbadi et al., 2021) suggests that -in fact-experiments have a pivotal role in bridging sustainable mobility solutions and policy making. (Newton and Frantzeskaki, 2021) also highlight the importance of experimentation for innovation at urban level, while (Hughes et al., 2020) underline the contribution of pilot projects in preparing the ground for innovation in urban climate policy overall. The strength of the link between demonstration projects and policy implementation may be explained by the increase in both effectiveness and user acceptance of measures that have been already tested in pilots prior to a large scale implementation (Liu and Van de Walle, 2022). Therefore, it may be reasonable to expect that cities considering themselves as prepared to address the climate neutrality challenge have been already involved in research and innovation activities that explored the applicability of new solutions.

Research and innovation activities are often identified as fundamental for the transition to climate neutrality (Huovila et al., 2022; Maestosi et al., 2021). Urban transport policy faces additional challenges due to the multifaceted nature of cities and the inherent complexity of mobility, which both require tailored responses (Lindholm and Behrends, 2012; Macharis and Kin, 2017; May, 2015). In such a context, relevant research and innovation activities may not be strictly limited to technologies for reducing transport emissions. The ‘twin’ green and digital transition can complement the ambitions for urban climate neutrality, adding further policy objectives and potential instruments (Bianchini et al., 2023; Husain et al., 2022).

In the work presented here, we combined the information provided by the 362 candidate cities of the EU Mission with detailed data on research and innovation activities at city level across the EU. The methodology that we applied addresses two key research questions:

• What is the contribution of transport research and innovation activities in the preparation of cities for climate neutrality?
• How is research and innovation in specific transport technologies relevant to a city's ambition for climate neutrality?

The underlying hypothesis is that the candidate cities are representative of EU cities that have the ambition and the capacity to reach climate neutrality by 2030. We first explore the importance and describe the general context of transport policy in the candidate cities. As a second step, we test the hypothesis that the cities' perceived preparedness for the EU Mission is correlated with their proximity to clusters of (transport) research and innovation. Finally, we compare how research and innovation activities in different thematic areas of transport may influence the (perceived) preparedness of the cities. The overall objective is to determine whether a track record in transport research and innovation is an influencing factor for a city's determination to undertake the transformation required to move to a climate-neutral future. We explored how previous experiences in R&I programmes (notably, Horizon, 2020) affect or correlate with the city ambition and an evaluation of the contribution of specific transport technologies and roadmaps, in particular related to smart transport. The analysis is directed at:

i) urban and transport planners and scholars, as it identifies how and which research strands in transport have concretised in ambitious climate targets, what research areas and topics are prominent in ambitious city clusters, and what geographical areas remain underserved,
ii) cities, as it aggregates the insights gathered through a number of European Commission datasets (not all publicly available) to distil clear priorities and multiple narratives on the link between R&I and deep transport decarbonisation and to inform future policies and strategies, and
iii) regional and national governments as it includes a territorial characterisation pointing towards the benefits of an effective multilevel governance approach.

## 2 Data and methods

**Fig. 1:**
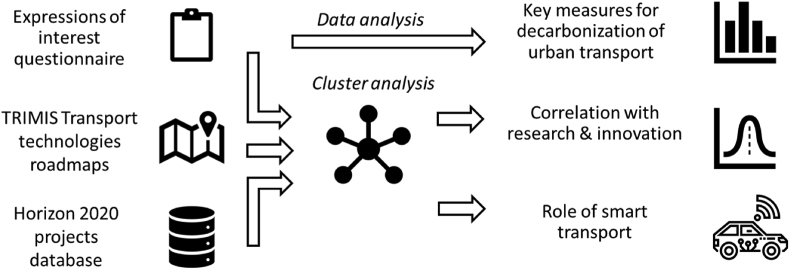
Schematisation of the methodological approach.

The methodology we followed combined three sources of data (discussed in detail in the following sub-sections), a number of standard statistical analyses, and a tailored clustering approach (Fig. 1).

The expressions of interest of the 362 candidate cities provide detailed information on the cities’ profiles and policy priorities and allow geographic information to be extracted to map the spatial distribution of the candidate cities. The analysis of the responses to specific questions reveals the importance of transport within urban climate policy and outlines the main elements of urban transport policy in candidate cities.

The CORDIS database contains project-level information for all activities funded by the EU's framework programmes for research and innovation. We use the data corresponding to the Horizon 2020 (H2020) programme as an indicator of the research and innovation intensity in each city by aggregating the funding received by each project partner located in each city across the EU. This permitted the mapping of the geographic distribution of H2020, which was consequently used for the exploration of the spatial correlation between candidate cities and clusters of H2020 activity.

The TRIMIS database allowed an additional level of analysis. TRIMIS identifies whether a specific project addresses transport issues and-for those that do-tags the specific transport technology areas that they address.

Combining the project level information from TRIMIS with the funding and location information included in CORDIS for all partners involved in the projects allowed us to detect and rank clusters of research and innovation across the EU. Such clusters, at either total H2020 level or specifically for transport related issues, were identified using a clustering algorithm that took into account both activity levels and spatial aspects. Once such clusters were defined, we performed an Odds Ratio analysis to test the hypothesis of whether cities close to cluster of high research and innovation intensity have a higher probability of being a candidate city. We confirmed the findings with standard regression models. Finally, utilizing the detail on the transport technologies included in the TRIMIS database, we explored how a focus on specific transport technologies – especially smart transport-further influenced the probabilities.

### 2.1 EU mission questionnaire

The European Commission invited cities to state their interest in becoming climate-neutral by 2030 and to submit information on their current situation, ongoing work, and future plans. The call received 362 eligible expressions of interest: 314 from cities within EU-27 and 48 from cities from Associated Countries (or in the process of negotiating association) to Horizon Europe (Turkey, United Kingdom, Norway, Israel, Albania, Iceland, Montenegro and Bosnia-Herzegovina). The questionnaire consisted of 374 mixed-type questions (single choice, multiple choice, free text, file upload) and provided the basis for a systematic assessment of the cities' preparedness, commitment and capacity to reach climate neutrality by 2030. It was divided in 9 thematic sections that covered information concerning eligibility criteria, additional city information, current emissions and policies, level of ambition, partnerships, investment plans, governance and barriers (Vetters et al., 2021). Based on these questionnaires, the European Commission evaluated the ambition, preparedness and potential impact of each candidate's proposal and selected 101 EU cities (2 cities expressed interest as a group) and 12 additional cities coming from countries associated or in the process of being associated to Horizon Europe (European Commission and Directorate-General for Research and Innovation, 2022).

Previous analyses of the expressions of interest investigated in detail the city perspective on emissions accounting and offsetting (Ulpiani et al., 2023c), on the risks associated with a fast decarbonisation process (Ulpiani and Vetters, 2023), on the relevance of embedding climate justice principles in the design of climate neutrality (Della Valle et al., 2023), and on the funding and financing architecture that may be necessary ((Ulpiani et al., 2023a)). Furthermore, a set of “sectoral” studies examined the role that buildings (Ulpiani et al., 2023b), renewable energy sources (Ulpiani et al., 2023d), and circular economy (Möslinger et al., 2023) would play in the realisation of climate neutrality. This study complements the picture by looking at the transport sector and how its decarbonisation is deeply rooted in research, development and innovation. The main objective is to profile the 362 candidate cities (as representative of EU cities that have the ambition for climate neutrality) and evaluate the role of transport policy as well as the relationship of transport research, innovation and development in committing to climate neutrality. We also present results specific to the 101 EU cities that were selected, mainly for comparison purposes. We added the geographic coordinates of each city to the dataset in order to perform geospatial analyses, and we used standard frequency statistics for the analysis of the responses. A subset of questions is used for the analysis, investigating:

• GHG emissions reduction target for the future and the associated sectors: cities could state their official climate mitigation targets already in place and their adopted plans in terms of climate change mitigation at sector or cross-sectoral level
• areas addressed by current transport policy: cities were asked to identify the policy areas presently tackled from a list of possible options that have relevance to climate neutrality;
• key measures put in place in the past directly or indirectly impacting emissions in the transport sector;
• any participation (and degree of involvement) in relevant initiatives, R&I projects, and competitions at EU, national or local level;
• digital solutions and smart city paradigms applied to the transport sector.

### 2.2 CORDIS database

The 10.13039/501100006221Community Research and Development Information Service (CORDIS) is the European Commission's primary source of results from the projects funded by the EU's framework programmes for research and innovation. CORDIS provides a public repository with detailed project information. We use project and participant information from the Horizon 2020 Framework Programme (H2020). H2020 was the EU's research and innovation funding programme from 2014 to 2020 with a budget of nearly €80 billion. H2020 was the successor of a series of 7 “Framework Programmes for Research and Technological Development”, the multi-annual frameworks that finance collaborative research and development activities in the EU. The 1st Framework Programme (FP) was launched in 1984 and the 7th lasted from 2007 to 2013. The general objective of H2020 was to help build a society and an economy based on knowledge and innovation, to provide funding for research, development and innovation, and to contribute towards the target of spending 3 % of EU gross domestic product (GDP) on research and development. H2020 marked both an increase in total funding and an extension of the scope of the FPs. It combined support to science and technology policy research, and spearheaded R&D integration and implementation (Varela-Vázquez and González-López, 2020). Participation in H2020 projects can be used as an indicator of research and innovation activity at organisation or spatial level, since the programme was the largest source of funding at European level. The time period covered is sufficiently long to ensure that the most relevant activities that support climate neutrality policies conceived in 2022 are included.

For each project funded under H2020, the database includes a project summary, the specific sub-programme and call it belongs to and the contact information of project leaders and partner organisations. The combination of project and participant data allows a link between funding and geographic information to be established. All project participants have a unique organisation code and address, including geographic coordinates. For each specific project, the role, type of activity and funding of each participant is stated.

We used the CORDIS data as a baseline for the analysis of the geographic distribution of H2020 projects and participants. The geographic coordinates included in the dataset are used to perform a spatial correlation between the locations of the candidate cities and those of project participants. This allowed the clustering approach to classify areas according to their participation level in H2020.

The full dataset used is openly available at: https://data.europa.eu/data/datasets/cordish2020projects (European Union Publications Office, 2015).

The CORDIS database is extensively used in the literature for the analysis of clusters of innovation (Capone, 2014), the definition of technology roadmaps (Kotlík, 2018), the correlation with regional innovation (Varela-Vázquez and González-López, 2020) and the assessment of the policy relevance of research (Kropp and Larsen, 2022).

### 2.3 TRIMIS

The Transport Research and Innovation Monitoring and Information System (TRIMIS) provides open-access information on transport research and innovation. TRIMIS supports the implementation of transport policies of the 10.13039/501100000780European Union and at Member States level. TRIMIS analyses technology trends in the European transport sector (Tsakalidis et al., 2020a) and has been used in several analyses of research and innovation (Grosso et al., 2021; Tsakalidis et al., 2020b, 2021).

TRIMIS also participates in the development and monitoring of the Strategic Transport Research and Innovation Agenda (STRIA), which outlines transport R&I priorities to achieve a more sustainable European transport sector. In coordination with Member States and transport stakeholders, STRIA aims to set out common priorities to support and speed up the research, innovation and deployment process leading to radical technology changes in transport.

STRIA covers the following 8 thematic transport technology groups:

• Connected and automated transport, supporting the development of a customer-centric, intermodal and integrated transport system aiming at greater efficiency, safety and wellbeing and environmental impacts mitigation.
• Transport electrification, including new materials, advanced propulsion systems and Information and Communication Technology (ICT).
• Vehicle design and manufacturing, supporting the development of successful marketable transport vehicles with shorter development times.
• Low-emission alternative energy for transport, focusing on renewable fuels production, alternative fuel infrastructures, as well as the impact of these technologies on transport systems and services covering all transport modes.
• Network and traffic management systems, supporting the development of an advanced multimodal transport system through the optimization of the entire transport network across new areas.
• Smart mobility and services, supporting new and emerging technologies such as electric and autonomous vehicles, drone technology and on-demand mobility services.
• Transport infrastructure, supporting the development of research and innovation, testing new methodologies and preparing the ground for future transport infrastructure policies.
• Other research areas, not covered above.

In this analysis, we used the TRIMIS project database available at:

https://trimis.ec.europa.eu/projects-download/csv.

The TRIMIS database complements the other two datasets used, providing valuable details as regards projects specifically addressing transport and mobility. The first major advantage of TRIMIS is that it has already identified all H2020 projects –as well as many other research and innovation projects-that are relevant to the sector. Since it uses the same project IDs as the CORDIS database, comparing the two datasets allows for comparisons between overall and transport-specific H2020 funding at various geographic levels. In addition, TRIMIS associates each project to one or more STRIA roadmaps and transport mode. This information allows a deeper analysis of the candidate cities information by linking specific projects to technology roadmaps or comparing the relative weight of each roadmap in the set of measures applied in each city. We used the information included in TRIMIS, combined with the spatial data available in the CORDIS dataset, as input for the clustering analysis of research and innovation in transport research and innovation. In addition, we quantified the role of each technology roadmap through standard frequency statistics.

### 2.4 Identification of research and innovation clusters

A key step in our methodology was the exploration of the link between a city's access to research and innovation projects and its willingness to abate local emissions within the framework of the EU Mission. The hypothesis tested is whether a city's proximity to research and innovation activities increased its probability of being a candidate for the EU Mission. The rationale was that there is probably a correlation between the research and innovation context of a city and its levels of preparedness, commitment and ambition towards climate neutrality.

The candidate cities vary significantly in terms of their definition of administrative units and can represent single municipalities, groups of cities, or parts of larger metropolitan areas. The definition of a city is not uniform across the EU either. Cities may consist of several smaller administrative units or be grouped into a larger aggregation. A similar issue exists when province (NUTS3) or region (NUTS2) level information is used, with different levels and sizes used in each EU Member State. While both the information in the cities’ questionnaires and the CORDIS/TRIMIS databases include the city and region name, neither was suitable for a meaningful comparison.

We used a more flexible geographic method instead, that of spatial clustering based on the coordinates of both city candidates and project participants. The idea was that a candidate city-regardless of its administrative definition-can be considered as being part of a cluster that concentrates a significant number of projects. The clusters do not follow administrative definitions and depend only on geographic proximity. Such a formulation would allow, for example, labelling a candidate city that is part of a metropolitan area as part of a cluster of projects carried out by institutions located in other parts of the metropolitan area. It would also prevent the other extreme, the case of all municipalities belonging to a NUTS2 region to be considered as part of the same cluster.

We applied a well-known, density-based clustering algorithm, dbscan (Hahsler et al., 2019). Its main advantage compared to other frequently used clustering algorithms like kmeans (Lloyd, 1982) is that dbscan allows the definition of different cluster sizes, as well as the identification of outliers that do not belong to any cluster. This is a useful attribute for this analysis, since it prevents cities in low research and innovation intensity areas from being allocated to the closest cluster. The algorithm requires two parameters: *ε (eps)*, which is the maximum distance between two points belonging to a cluster, and *minPts*, the minimum number of points required to form a cluster. Since the number and size of clusters can affect the robustness of the analysis, we explored a range of parameter values in order for the resulting clusters to allow for interpretable results. The cluster definition is based on the following conditions (Ester et al., 1996):

Eps-neighbourhood of an object x is denoted by

(Eq. 1) $$N_{\varepsilon }\left(x\right)=\left\{\;y\;|\;dist\;\left(x,y\right)\leq \varepsilon \;\right\}$$

An object y is considered as directly density-reachable from an object x if y is part of the neighbourhood of x:

(Eq. 2) $$y\;{\in N}_{\varepsilon }\left(x\right)$$

and x is already part of a cluster that satisfies the condition of minimum number of points:

(Eq. 3) $$\left|N_{\varepsilon }\left(x\right)\right|\geq minPts$$

In addition, two objects x, y are considered as density connected if there is an object z such that both objects are density-reachable from z.

A cluster C is a non-empty subset of objects satisfying:

1) ∀x,y: if x∈C and y is density-reachable from x, then x∈C.
2) ∀x,y∈C: x is density-connected to y.

We used an iterative process that aimed to identify the combination of parameters that led to a number of clusters between 400 and 500, and a median of the distance of each cluster centre to its closest neighbour between 50 and 100 km. The parameters allowing clusters that met these conditions were *eps* = 0.15 as the maximum distance from a cluster member and *minPts* = 10 as the minimum number of points in a cluster.

The CORDIS database includes 8769 unique EU-27 cities with at least one participation in a H2020 project. Applying the clustering approach on all unique cities led to the identification of 472 individual clusters. We used the total funding of projects belonging to each cluster as a measure of the research and innovation intensity in the cluster. This was done both for the full H2020 programme (using CORDIS data) and for projects addressing transport and mobility issues (using TRIMIS data). For each cluster $C_{j}$, the total funding $F_{j}^{H}$ for project participants within the cluster is:

(Eq. 4) $$F_{j}^{H}=\sum_{i\epsilon C_{j}}F_{i}^{H}$$

where H corresponds to either the full H2020 programme, or to specifically transport and mobility projects in H2020, and $F_{i}^{H}$ is the total funding received by participants located in each city i which belongs to cluster $C_{j}$.

We grouped the 472 clusters in terciles based on the total project funding in the cluster, which would correspond to three levels of intensity (high, medium, low) for either the full H2020 programme or the subset of transport and mobility projects.

Based on these, we estimated the Odds Ratios for having expressed interest in the EU mission among cities belonging to each of the three levels of intensity:

(Eq. 5) $$r_{q}^{H}=\frac{p\left(i\;\epsilon \;M\;|\;i\;\epsilon \;Q_{q}^{H}\right)\;/\left(1-p\left(i\;\epsilon \;M\;|\;i\;\epsilon \;Q_{q}^{H}\right)\;\right)}{p\left(i\;\epsilon \;M\;\left|\;i\;\epsilon \;Q_{q=1}^{H})\;/(1-p(i\;\epsilon \;M\;\right|\;i\;\epsilon \;Q_{q=1}^{H})\;\right)}$$

Where M is the set of candidate cities in EU Member States (n = 314) and $Q_{q=1,2,3}^{H}$ denotes the set of cities that belong to each tercile for each H aggregation level (with 1 corresponding to the lowest tercile and 3 to the highest). The probabilities were calculated using the full set of cities (i) included in CORDIS (n = 8769).

We repeated the calculation of Odds Ratios in order to explore whether the probability for a city being selected for the EU Mission was also correlated with the level of intensity. In this case, M corresponded to the set of 101 cities that were eventually selected by the European Commission.

As an additional step, in order to confirm the impact of participation in H2020 as revealed by the Odds Ratios, we applied a standard linear regression model at cluster level:

(Eq. 6) $$p_{j}=f\left(F_{j}^{H},{\bar{GDP}}_{j},{\bar{CO2}}_{j}\right)$$

where $p_{j}$ is the share of the cities belonging to cluster j that were candidates for the EU Mission, ${\bar{GDP}}_{j}$ the mean of GDP per capita within cluster j, and ${\bar{CO2}}_{j}$ the mean of emissions of CO_2_ per capita within cluster j.

Finally, we used the STRIA roadmap information included in TRIMIS to explore the correlation with specific transport technologies. For each cluster, we calculated the number of projects belonging to each of the 8 technology groups covered in STRIA. This indicator allowed us to explore whether higher project activity in any of the 8 technology groups increased the probability of a city being a candidate.

## 3 Results

**Fig. 2:**
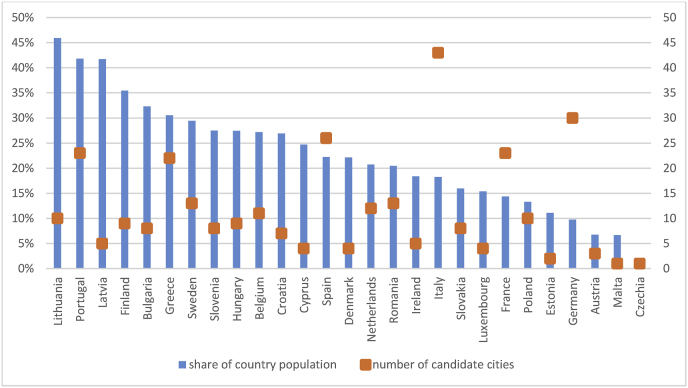
Number of candidates and corresponding share of population by EU Member State.

The 362 questionnaires submitted by cities suggest that there is a high level of interest in the EU Mission. The 314 candidate cities from the EU-27 account for a remarkable 18% of total population, with a large variety among candidate cities as regards size, location and typology. Nevertheless, no discernible pattern can be recognised in terms of city or country characteristics. Apart from the country size and the degree of urban concentration that can explain some of the exceptionally high or low values in some countries, there is –in principle-no indication of economic, environmental or political factors influencing a city's choice to be a candidate (Fig. 2). The levels of preparedness, commitment and ambition towards climate neutrality are probably themselves main driving factors for the cities and depend –to a large extent-on each city's policy track record. In the following sub-sections, we explore the measures applied by candidate cities in order to identify possible common patterns. Assuming that the cities that consider themselves as prepared to take on the challenges of the EU Mission are a representative sample of the front-runners, such patterns can provide guidance on promising measures towards achieving climate neutrality. Transport and mobility have a crucial role in policies towards climate neutrality, and the specific measures applied at city level can be useful in formulating urban transport and mobility strategies. Our analysis also identifies a strong relevance of a city's proximity to clusters of R&I activity, which appears to be a factor that affects how prepared it is for the application of innovative measures. Finally, specifically for transport and mobility, we explore the role of different technology roadmaps in forming the cities' strategies.

### 3.1 Key measures towards achieving carbon neutrality

**Fig. 3:**
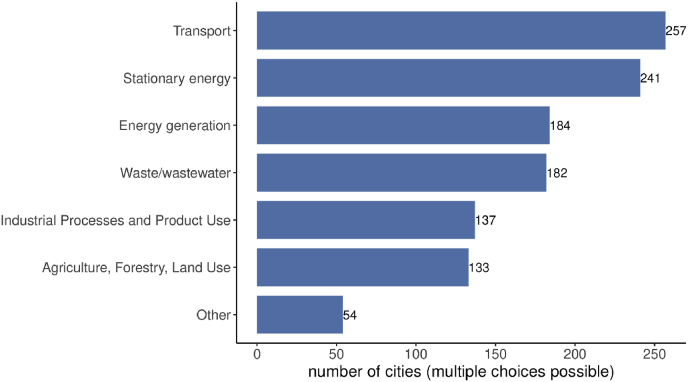
Sectors covered by the emissions reduction target for the future (N = 263).

The information provided by the candidate cities in the questionnaires aimed at demonstrating that they are prepared for the climate neutrality challenge and have identified the policies and measures to achieve the EU Mission goals. We assume that this information reflects the policy mix that each city considers as the most suitable, and that the analysis of the questionnaires at aggregate level can provide a representative picture of the strategies followed by cities with a climate neutrality ambition. The majority of the candidate cities have already adopted a GHG emissions reduction target for the future (266 out of 362, 73.5%) and have identified the relevant sectors of intervention (263 out of 362, 72.7%). Transport has a visibly central role, flagged by 97.7% of these cities (257 out of 263). Stationary energy -representing the energy consumption associated with buildings, equipment, facilities and public lighting-is also close in importance. Since the priorities and the profiles of the applicant cities differ significantly, the other main sectors responsible for GHG emissions vary in terms of their relative weight. Energy generation is a priority mainly for cities with generation activities within the areas covered by the targets. Waste and wastewater treatment are addressed primarily in larger urban areas. A further segmentation can be observed as regards industrial and agricultural sectors, where a concentration of such activities in the city area raises the importance of either sector as a GHG source (Fig. 3).

**Fig. 4:**
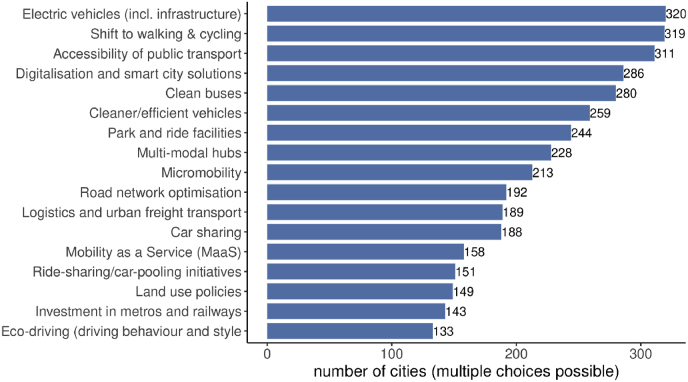
Areas addressed by city's current transport policy (N = 356).

Elaborating on the transport sector, the majority of candidate cities already applies a policy mix that intends to combine the goal of decarbonizing transport with the numerous other dimensions of climate neutrality and efficient mobility at urban level (Fig. 4). Technological solutions, especially in terms of electric vehicles and infrastructure, emerge as a top priority (320 out of 356 cities that answered the question, 89.9%), closely followed by interventions conducive to modal shift to active, greener modes such as walking and cycling (89.6%) or public transport (87.4%), but also by digital and smart city solutions (80.3%). Several other areas appear as part of the cities' current transport policy mix. The scope of targeted areas indicates that a climate-neutral urban transport policy requires a combination of a wide range of technological and planning solutions, which depend on each city's priorities, capacities and competences. It also suggests that the cities that are ambitious enough to aim for climate neutrality are also willing to experiment with emerging technologies in order to explore ways to improve their urban transport system.

**Fig. 5:**
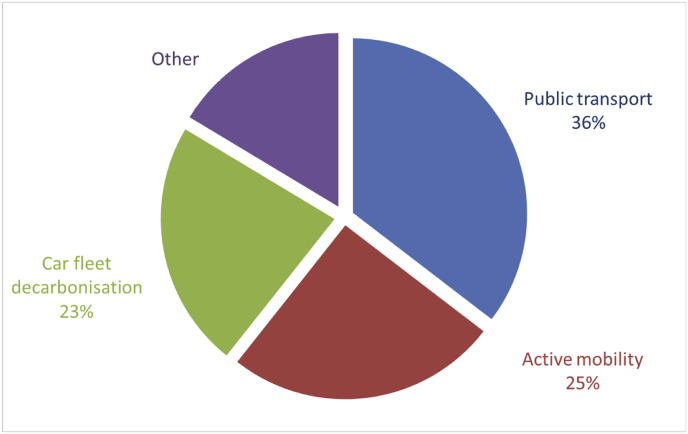
Main categories of reported key measures addressing transport issues.

Cities were also asked to describe, in free text, up to 5 key climate change mitigation/GHG reduction measures that they have successfully implemented since 2005. Key measures addressing specifically transport and mobility were reported by 212 cities, with a total of 305 measures described (Fig. 5).

The largest group of reported key measures is public transport (108 measures). Out of these, technological measures focusing on the conversion of the public transport fleets into electric and other alternatives (e.g. hydrogen or bio-gas) are –or have been-successfully implemented in 62 cities. Infrastructure measures focusing on increasing the supply, accessibility, quality of service and –in general-attractiveness of public transport were reported in 41 cases, 31 at city-level measures, 3 at district or smaller scale, and 7 a larger than city level.

### 3.2 Role of research and development

**Fig. 6:**
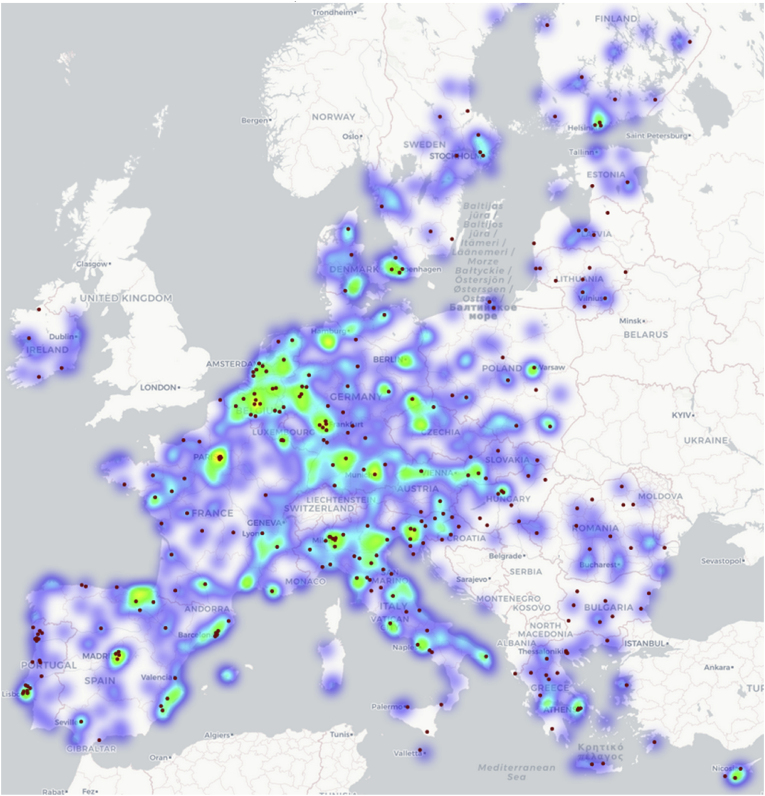
Heatmap of project participants in H2020 projects related to transport and mobility and location of EU-27 candidate cities (red points). (For interpretation of the references to colour in this figure legend, the reader is referred to the Web version of this article.)

Juxtaposing the locations of the 314 candidate cities from the EU with the heatmap of the locations of participants in H2020 projects related to transport and mobility suggests that there is a high correlation between a city's proximity to research activities and its being a candidate for the EU Mission (Fig. 6). While this is an interesting observation as a starting point, it is still not sufficient to establish a causal relationship since it does not take into account the spatial distribution of cities that were not candidates for the EU Mission and does not allow the comparison with their respective proximity.

In order to make discard the possibility of this pattern being simply the result of spatial auto-correlation, we constructed the counterfactual by utilizing the data available in the CORDIS and TRIMIS databases. We followed the clustering approach described in Section 2.4 to identify 472 clusters of research and innovation activity and group them into 3 levels that depend on total project funding. We allocated the 8769 unique EU-27 cities that appear in the CORDIS dataset to their respective cluster and flagged whether they are one of the 314 candidate cities from the EU.

**Fig. 7:**
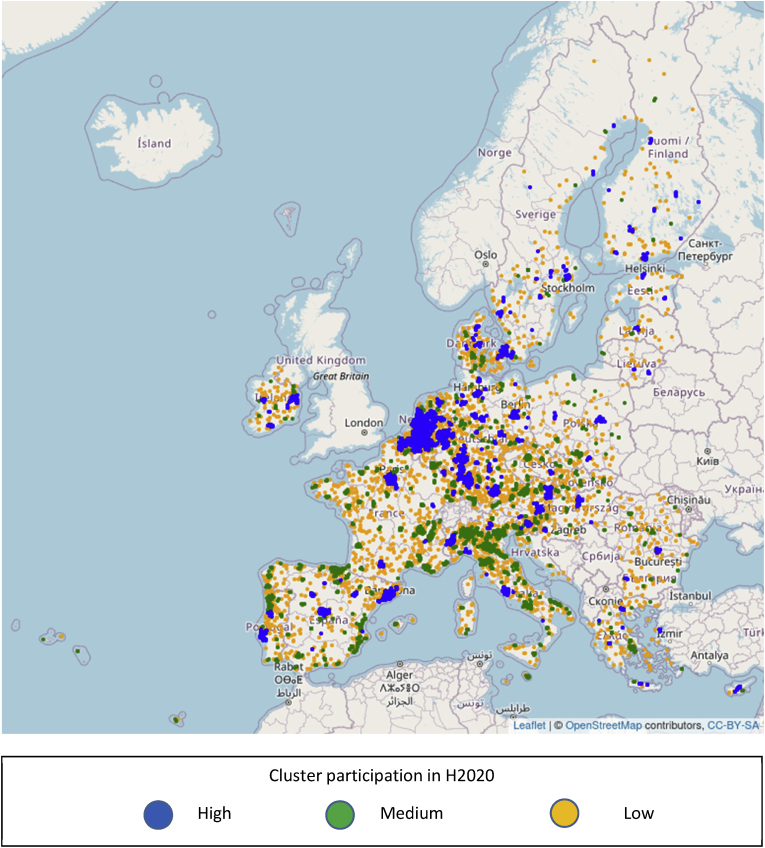
Geographic distribution of 8769 EU cities with presence in H2020 and level of participation of their corresponding cluster in H2020 projects.

Fig. 7 maps all 8769 cities and classifies them according to whether they belong to a cluster of low, medium or high intensity on H2020 participation, using the cluster's total funding as a measure. The map reveals a pattern of high disparity in terms of concentration. Extensive geographic areas of high intensity visible in the Netherlands, Belgium and a large part of Germany; local foci around main urban areas in Austria, Denmark, France, Ireland, Portugal and Spain; strips of medium intensity in parts of Italy, Portugal and Spain; and wide areas with isolated clusters or clusters with low intensity.

**Fig. 8:**
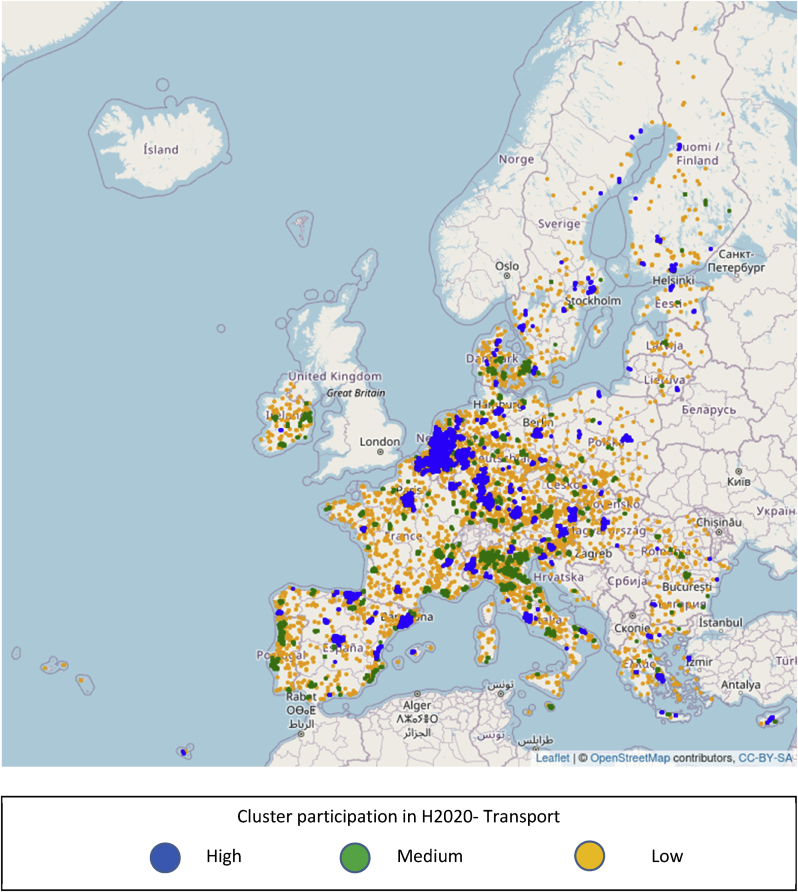
Geographic distribution of 8769 EU cities with presence in H2020 and level of participation of their corresponding cluster in H2020 projects addressing transport issues.

In Fig. 8, the mapping is repeated using the funding of H2020 projects specifically addressing transport related issues, using the information included in TRIMIS. A high level of disparity is still present, but the patterns of spatial concentration of activity appear to have changed. Clusters in Denmark, Ireland and Portugal have in general a lower intensity as regards transport projects compared to overall H2020 projects, signifying that the relative weight of transport activities within H2020 is lower than the average in these countries. On the other hand, new regional clusters of high intensity appear, as for example in the north and east of Spain or in Greece. In such cases, the relative weight of transport related activities within H2020 would be higher than the average.

**Fig. 9:**
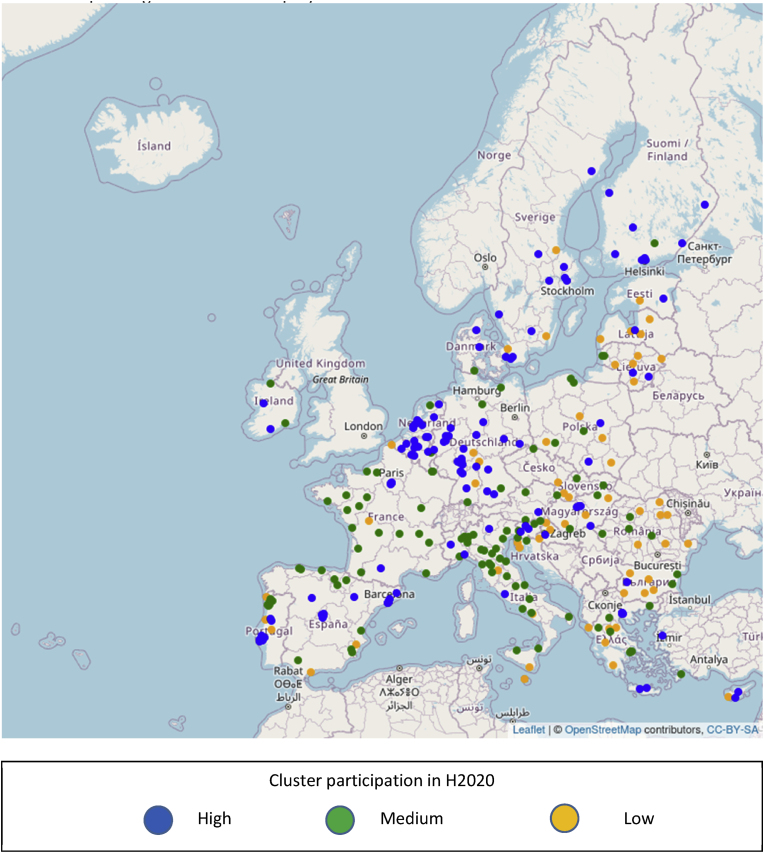
Geographic distribution of 314 candidate cities from the EU and level of participation of their corresponding cluster in H2020 projects.

**Fig. 10:**
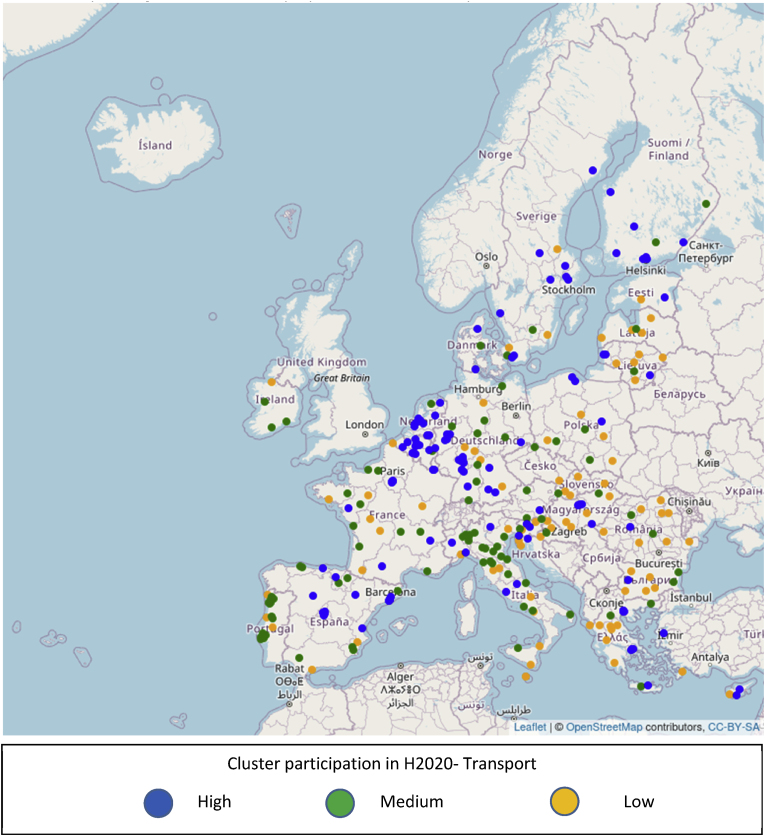
Geographic distribution of 314 candidate cities from the EU and level of participation of their corresponding cluster in H2020 projects related to transport.

The differences observed in the spatial concentration of H2020 activity compared to that of H2020 projects specifically addressing transport (Fig. 7, Fig. 8) are also reflected in the distribution of the candidate cities to clusters of different participation levels. The corresponding Fig. 9, Fig. 10 suggest that if a candidate city belong to a high participation cluster for H2020, it does not necessarily belong to a high participation cluster for transport also, or vice versa. For example, Valencia, Spain is a member of cluster of medium participation in H2020, which we qualify as of high participation if only transport related projects are taken into account. Conversely, the cluster around Lisbon is in the tercile of high participation as regards H2020, but that of medium participation (2nd tercile) as regards transport projects. The differences in terms of cluster participation are visible in Fig. 9, Fig. 10.

The information that is visualized in Fig. 7, Fig. 8, Fig. 9, Fig. 10 also allows the construction of two matrices that summarize the number of cities in each cluster type for candidate and non-candidate cities. In addition, even though the selection criteria for the 100 cities covered a wider range of issues, we added a column with an indication of how many cities from each tercile of clusters were selected for the EU Mission.

Table 1 describes the distribution in clusters according to funding in H2020 as a whole, and Table 2 according to funding in projects relevant to transport and mobility within H2020.

**Table 1:** Summary statistics of clusters, full Horizon 2020 programme.

		Candidate for EU Mission		Selected
		No	Yes	
Cluster participation in Horizon 2020	Low	2650	65	11
	Medium	2974	122	35
	High	2831	127	55

**Table 2:** Summary statistics of clusters, projects related to transport in Horizon 2020.

		Candidate for EU Mission		Selected
		No	Yes	
Cluster participation in transport/Horizon 2020	Low	2816	87	16
	Medium	2743	109	34
	High	2896	118	51

**Fig. 11:**
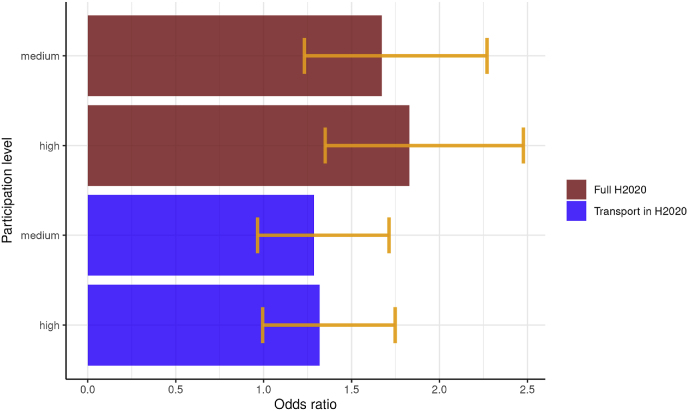
Odds Ratios of city being a candidate for EU Mission depending on proximity to research and innovation activity clusters (95% level of confidence).

We calculated the Odds Ratios for cities belonging to clusters with high or medium participation compared to those in low participation clusters, for both overall (Table 1) and transport-specific (Table 2) projects and summarized the results in Fig. 11.

Cities belonging to a cluster of medium intensity as regards funding in the overall Horizon programme are more likely to be a candidate for the EU mission than cities belonging to a low intensity cluster, with a mean Odds Ratio of 1.67 (within a 95% confidence interval of 1.23–2.27). Those belonging to a high density sector have a visibly higher Odds Ratio mean of 1.83 (1.35–2.48). These ratios suggest that there is a certain effect of proximity to H2020 activities.

When transport and mobility projects are concerned, cities in medium intensity clusters are on average 1.29 times more likely to be candidates than cities in low intensity clusters (0.97–1.71). The effect is marginally stronger for cities in high intensity clusters, which have on average 1.32 times the odds of cities in low intensity clusters (0.99–1.75).

The results reinforce the conclusion that there is a strong effect of proximity to research and innovation activities. Cities that form part of a cluster with high levels of participation in H2020 have higher odds of being a candidate for the EU Mission than cities that belong to clusters with low or medium participation. A similar, though weaker, effect can be observed for projects specifically addressing transport within H2020. This suggest that cities that have a track record in H2020 participation may have been better prepared to be a candidate for the EU Mission. Nevertheless, while transport is an important part of urban climate policy, it is evident that a wider focus of research innovation activities is probably more advantageous than one with a higher weight given to transport issues.

**Fig. 12:**
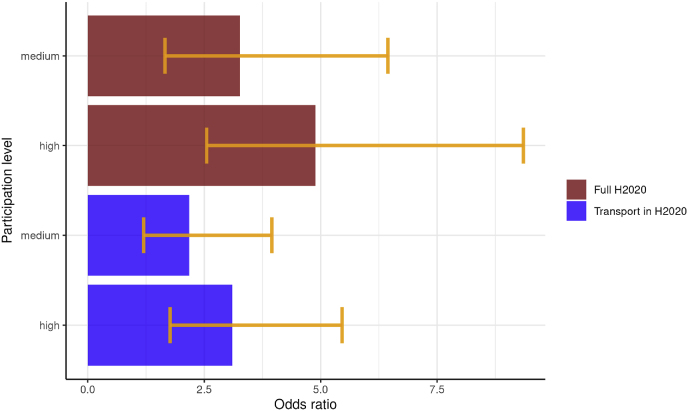
Odds Ratios of city being selected among the 100 EU Mission cities depending on proximity to research and innovation activity clusters (95% level of confidence).

We extended the Odds Ratios analysis to compare how participation in H2020 affects the odds of a candidate city being selected among the 100 EU Mission cities. While the selection criteria cover a much wider spectrum of issues and sectors than those addressed here, proximity to clusters of high H2020 participation still appears to have an important role. Cities belonging to a cluster of high H2020 participation have –on average - 4.89 times higher chances than the ones belonging to a cluster with low participation. Clusters with high participation in transport specific projects also have a positive influence, with 3.11 times higher chances than those with low participation (Fig. 12).

In addition to the above, we explored two regression models to confirm the observation concerning the influence of H2020 participation on the probability of a city being a candidate to the EU Mission, as described in Section 2.4. The dependent variable is the ratio of candidate cities to total number of cities for each of the 472 individual clusters. The total funding received by participants within a cluster, both for the H2020 as a whole (Table 3) and for H2020 projects addressing transport issues specifically (Table 4) appears to be statistically significant. Average GDP per capita and CO2 emissions per capita have a negative coefficient, though not at a statistically significant level, an observation that suggests that cities with higher GDP per capita or higher CO2 emissions per capita do not have a higher probability of being a candidate for the EU Mission.

**Table 3:**
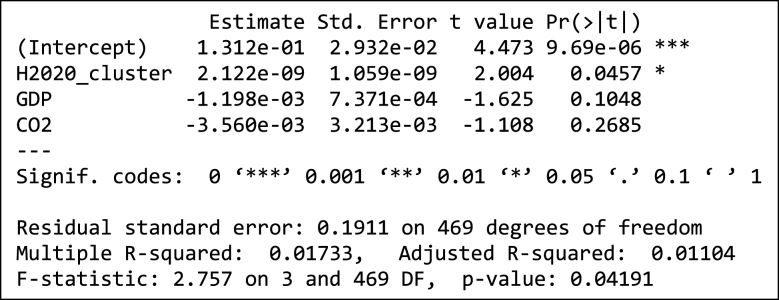
Regression model of share of cluster candidates depending on H2020 participation.

**Table 4:**
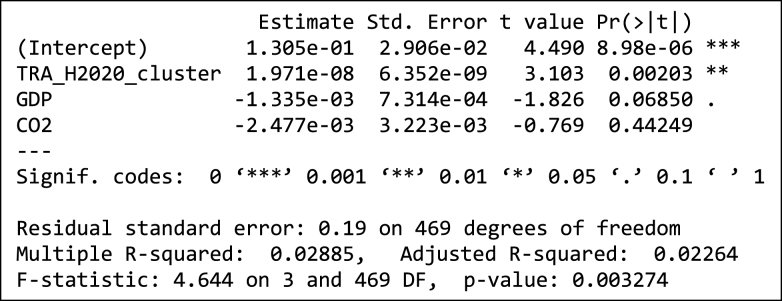
Regression model of share of cluster candidates depending on H2020 participation (transport only).

**Fig. 13:**
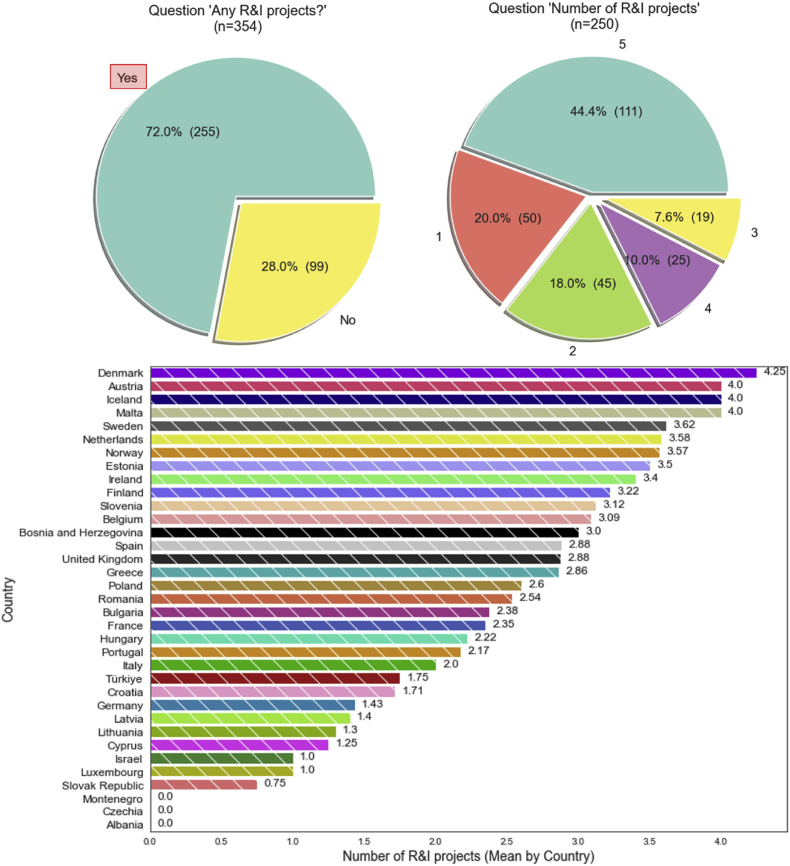
Number of R&I projects relevant to climate change mitigation/GHG emissions reduction and breakdown by country.

The information provided by the cities in the questionnaires confirms the importance of research and innovation for the candidate cities. Participation in European R&I projects since 2005 is widespread, with 255 out of 354 reporting at least one project (Fig. 13). The majority (44.4%) of the 250 cities that declared a specific number of existing R&I projects reported 5 of them (the maximum allowed). Nevertheless, the average number of projects of candidate cities by country shows high variability, mostly due to the low number of candidates in some countries.

**Fig. 14:**
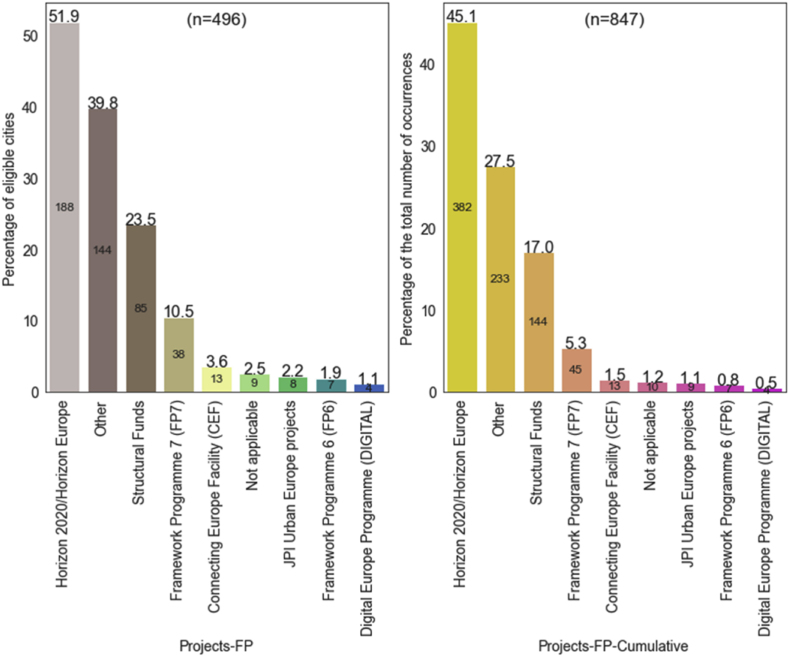
R&I projects and their framework programmes: variety and popularity.

The distribution of projects by specific R&I programmes also confirms the high relevance of the H2020 programme (Fig. 14). The left side of the figure reflects the variety of Framework Programmes in the portfolio of candidate cities as it excludes repetitions (same Framework Programme for same city across different projects), whereas the right side accounts for all answers provided, thus revealing which Framework Programme are more popular overall. Horizon 2020/Horizon Europe dominates the type of EU R&I projects reported by cities (188 cities mentioned it at least once, covering 45.1% of the total occurrences), followed by ‘Structural Funds’ and FP7 projects. ‘Connecting Europe Facility’ (CEF) projects, ‘Joint Programing Initiative’ (JPI) Urban Europe projects, FP6 projects, and DIGITAL projects are comparatively much less popular (reported at least once by 32 eligible cities in total). The category ‘Other’ was frequently selected (233 occurrences, 144 cities having selected it at least once) demonstrating a much larger pool of project-based programmes cities have familiarised with in tackling GHG emissions reduction or climate change mitigation compared to those proposed in the answer options.

The keywords extracted from cities' free text descriptions can be also indicative of the scope of the R&I projects they participated in. The most frequently appearing terms include ‘energy’ (87 instances), ‘sustainability’ (53), ‘smart and digital (51 + 12), ‘climate’ (37), ‘mobility’ & ‘transport’ (34 + 20), ‘greening’ (30), and ‘circularity’ (23).

### 3.3 Smart mobility

**Fig. 15:**
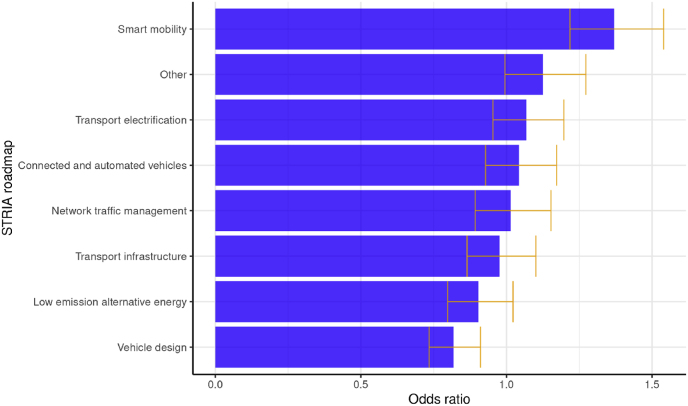
Odds Ratios of city being a candidate for EU Mission depending on projects by STRIA technology roadmap.

In order to explore the relevance of specific transport technology themes for the candidate cities, we associated the H2020 projects present in each cluster with one or more of the eight STRIA technology roadmaps. We then compared the frequency of each technology roadmap in clusters with candidate and non-candidate cities, in order to explore whether a specific roadmap has a higher probability of appearing in the clusters of candidate cities. Comparing the resulting Odds Ratios per technology roadmap suggests that there is a visible effect of the focus of R&I activities. Cities in clusters with a high presence of projects matching specific technology roadmaps have different probabilities of being candidates (Fig. 15).

Starting from the lowest odds ratios, cities in clusters that have projects addressing vehicle design (mean odds ratio of 0.82) or low emission alternative energy (0.90) appear as having a lower probability to be candidates than cities in clusters that do not. A possible explanation for this can be that such technologies have a global impact and do not necessarily influence urban policy towards climate neutrality in the local area. A similar reasoning can be used for the next four roadmaps: transport infrastructure, network traffic management, connected and automated vehicles and transport electrification R&I activities appear at comparable levels of frequency in clusters with or without candidate cities (mean odds ratio values between 0.98 and 1.07). Such technologies may have a more local focus, but still do not appear to be a differentiating factor between candidate and non-candidate cities.

In contrast, smart mobility projects appear to have a clear influence on the cities’ probabilities to be a candidate for the EU Mission. Cities in clusters with projects addressing smart mobility issues are 1.37 more likely to be a candidate than cities in clusters that do not (with a 95% confidence interval of 1.22–1.54). Using the same rationale as before, this may indicate that smart mobility projects have a higher relevance for urban areas as regards their preparedness for the EU Mission. In a similar fashion, projects addressing other issues outside the seven thematic STRIA maps appear to have certain relevance, with an odds ratio of 1.13 (with a 95% confidence interval of 0.99–1.27). Projects in this category address issues that are not linked to the main technology themes covered by STRIA and probably have a stronger urban transport policy dimension.

The projects flagged as addressing smart mobility in STRIA cover five main areas (Dotter et al., 2019):

• smart mobility, sustainable land use, active mobility
• integration of smart mobility with public transport services and zero-carbon energy systems
• digital infrastructure and mobility data management
• intermodality and interoperability
• automated, air and virtual mobility

To compare with the information provided by the candidate cities, we used the sub-section of the questionnaire that addressed digital solutions and smart city paradigms. Cities were asked which elements they had in place to enable or incentivise digitalisation and smart city solutions intended to support the transition towards climate neutrality. Policies and strategies could be either standalone or part of broader urban/innovation/sustainability strategies/policies. Digitalisation and smart city strategies and solutions were both very common answers, with more than 70% of the candidate cities reporting that these elements are already in place.

As regards specific solutions, 253 cities indicated that they implement ICT infrastructure to enable smart-city solutions. Some of the recurring infrastructure elements are edge computing, fibre optic networks, LoRaWAN, 5G, and integrated urban data platforms. 206 cities indicated that they use Internet-of-Things technology. The most mentioned applications are environmental monitoring (e.g., air quality, temperature and humidity; rain fall), traffic, mobility and parking management, smart and connected public lighting, waste management (e.g., smart bins), energy consumption, water management (e.g., monitor consumption and detect leaks), and crowd monitoring. 149 cities indicated they use open standards by preference and 96 cities indicated that they have experience with digital twins.

There is a strong R&I aspect in most of the smart city solutions presented by the candidate cities, which explains -to a large extent-the link with H2020 projects. Most of the involved technologies are still in early application phases and local pilots and demonstrations have been essential for their development. On the other hand, the cities that were directly involved most probably gained experience with such technologies and took them up as part of their portfolio of measures towards climate neutrality.

## 4 Discussion

Transport measures form part of the policy mix towards climate neutrality in the majority of the candidate cities for the EU Mission. This reflects both the importance of transport and mobility as a source of GHG emissions and the degree of competence of city authorities to address the sector at urban level. In a comparable assessment of 124 European cities participating in the voluntary agreement of the Covenant of Mayors (Croci et al., 2017), transport represented 26.3% of the total emission inventories. Given the ambition of the EU Mission for Climate Neutrality by 2030, it is indispensable that urban transport and mobility is included in city strategies.

Within the transport and mobility area, it is also evident that there is neither a single solution for climate neutrality, nor a policy mix that would suit all cases. Even though technological solutions are –arguably- readily available for a swift transition to carbon-free mobility options, their full-scale implementation still faces important obstacles in terms of infrastructure, funding, planning and user acceptance. The sample of the 362 candidate cities gives a representative picture of the policy mixes of front-runner cities. A common characteristic is that they apply a combination of measures that address emerging technologies with planning solutions. Most candidate cities include elements of promotion of public transport, active mobility, fleet decarbonisation and smart applications in their approaches. This is in line with international experience: the review in 29 major US cities identified modal shift to public and to non-motorized transport as a common element of climate action plans (Thomas A. Deetjen et al., 2018). Similar patterns can be seen in China (denHartog et al., 2018; Zhan and de Jong, 2018), Japan (Long et al., 2017) and Australia (Stanley et al., 2018).

Measures related to public transport are central in the policy mix since they serve further sustainable transport policy objectives apart from decarbonisation. Improved and extended public transport networks and services increase accessibility and reduce transport costs for residents and visitors. New public transport solutions have a pivotal role in stimulating policy processes for long-term transitions (Wimbadi et al., 2021). Likewise, measures that promote active mobility can improve quality of life and lead to health benefits, as also suggested in (Brand et al., 2021). Planning and digital solutions are also frequently used by cities as a means to facilitate the development of new urban models (Nieuwenhuijsen, 2021).

The results also suggest that the city size does matter, both in terms of preparedness (larger cities are more frequent in the candidate sample than in the overall distribution in Europe) and the suitable policy mix. This implies that there may be an advantage for larger cities in preparing for climate neutrality due to better access to resources, a finding also confirmed by (Otto et al., 2021; Reckien et al., 2018).

The findings concerning the importance of the various types of measures add to the general discussion on urban transport policy, confirming the relative importance of the main options and exploring the factors that affect the suitable policy mix for each particular urban area. Building on this initial analysis, the additional insight from this analysis is the evaluation of the role of research and innovation activity in preparing the city for climate neutrality. The link between proximity to clusters of R&I activities on transport and a city's willingness to be a candidate for the EU Mission can explain to a certain degree the geographic distribution of candidate cities, but also provides an important insight on the factors that may influence a city's preparedness. The higher odds for cities in clusters with high intensity in transport research suggests that transport is important for urban climate neutrality and that finding new solutions to challenges requires research and innovation. Cities that were active in the past -or have access to results and pilots-appear to be better prepared for the implementation of ambitious climate neutrality policies. An alternative reading would be that cities that realized the challenges early on also embarked in R&D activities. In either case, this observation confirms the hypothesis of climate policy leadership being path dependent, as suggested by (Otto et al., 2021). Today's leading cities in urban climate neutrality have been involved in climate policy development and research already since decades ago.

Within the spectrum of transport technologies, the category of smart urban mobility stands out as the one contributing the most to the differentiation of candidate cities in relation to other EU cities. Cities that consider themselves as capable of meeting the EU mission objectives are more likely to have access to research and innovation activities related to smart transport, indicating both the relevance of such application to the urban level and the importance of digital solutions in complementing other policy measures. The relevance of smart urban mobility solutions for the mitigation of GHG emissions confirms the findings of (Tran and Brand, 2021) in relation to smart urban mobility being an element of ambitious policy measures. Apart from increasing the efficiency of the transport system as a whole, emerging technologies can provide new sources of data and allow data collection and monitoring systems that can provide long-term evidence for strategic insights (Kandt and Batty, 2021). In a wider context, the importance of smart mobility may indicate the growing significance of the twin transition –green and digital-in the urban context. Cities possibly consider the growth of the digital economy as an opportunity for the development of sustainable development solutions, e.g. (Wang et al., 2022).

One of the many possible caveats that should be mentioned for the analysis presented here is that it is carried out from the perspective of urban transport policy. The data and results confirm the importance of the transport sector for climate neutrality at urban level, but it is evident that an integrated, multi-sectoral approach is required for cities to meet the EU Mission objectives. Another limitation may derive from the fact that the data reflect the views of the city authorities, which may be constrained by the scope of their own competences. This can explain why-for example-urban freight and logistics is an area that is not extensively addressed.

## 5 Conclusions

The 362 candidate cities for the EU Mission presented strategies for climate neutrality in urban transport that combine the introduction of new technologies -such as the provision of recharging infrastructure-with the promotion of public transport and active mobility to reduce car dependency. The information provided by the cities also confirms that urban planning solutions -such as extending the pedestrian areas, or applying emerging concepts like the 15 min city or megablocks-can influence mobility patterns and contribute to the reduction of transport activities and emissions. Nevertheless, the policy mix in each candidate city varies significantly, as a result of several factors, including differences in policy priorities, current emissions, city size, as well as financing and organisational capacity.

The complexity of urban climate policy revealed in the city questionnaires suggests that interventions in urban transport can be more successful if coordinated with a wider range of stakeholders and providers (e.g. transport operators, energy distributors). It is also important to take the potential limitations of a city's legislative power into account and actively involve institutions at the required administrative level (e.g. metropolitan or regional authority). New governance structures may be necessary in order to manage the path to decarbonisation. Given the diversity of city profiles and needs, there is not a single solution that is applicable to all urban transport systems (i.e. no ‘one size fits all’). Starting with small-scale solutions that are suitable to the local conditions can be a first step before scaling up to more ambitious or challenging options. New sources of data for transport (e.g. Internet of Things, sensors, mobile phones, social network data) can provide useful information for activity and mobility patterns that can be used for the optimization of urban transport management. The indirect benefits of decarbonizing urban transport, especially as regards congestion, air quality and health should be included in the evaluation of the interventions that target carbon neutrality.

Our results suggest that cities with a track record in transport research and innovation are more ambitious and better prepared for the challenge of climate neutrality. There is a strong link between a city's proximity to clusters that concentrate research and innovation activities and its probability to be among the candidate cities for the EU Mission. Within the transport field, many of the measures suggested had been previously tested in the context of EU funded projects. The data suggest that the commitment for climate neutrality is path dependent, with successful examples of candidate cities often coming from decades of dedicated policy development.

R&I activities appear to be a main ingredient in urban climate policy in general, but the effect is even more visible as regards transport and mobility. Innovative measures that support urban climate neutrality strategies can benefit from local pilots and demonstrations since they allow the evaluation of their applicability and can assist in raising user awareness and acceptance. Within the range of research areas related to transport, smart mobility appears to have a higher relevance for cities with climate neutrality ambitions, with numerous applications tested and implemented as a potential tool at city level. Urban climate policy objectives can be complementary to the challenges and opportunities raised by the ‘twin transition’, green and digital, especially as regards transport and mobility. R&I activities addressing aspects of transport policy related to the overall environmental, economic or social aspects of transport often support the transformation of the transport system in the direction of climate neutrality. The digital transition allows innovations that can reduce the carbon footprint of urban mobility by reducing transport demand, as well as by allowing the development of tools that increase overall efficiency.

Mission Cities are trailblazers in the realisation of net-zero futures, yet their experience and learning would be valuable and inspiring to mobilise any sub-national governments in the same quest. Beyond the critical role exerted by city networks in this regard, as part of the EU Mission, a Twinning Learning Programme has been launched to enable a peer learning framework (Net Zero Cities, 2023). In total, 40 cities from 20 countries have been selected to join the twinning programme in September 2023, with 27 of the selected Twin Cities being non-Mission cities. The next call for Twin Cities will open in early spring 2024. Twin Cities have been matched with 25 Pilot Cities activities, another Mission programme supporting cities to test and implement innovative approaches to rapid decarbonisation of all urban systems contributing to climate neutrality (including mobility, energy systems and the built environment, material and resource flows, natural areas, cultural/social/financial/institutional systems, and accessible public spaces). Together, cities will learn from each other, work across thematic areas and functional silos, and replicate systemic transformation approaches to accelerate towards climate neutrality by 2030. An additional proposal for the future could be to establish a similar collaboration framework for cities within the same region involving the regional government. This format could be critical in the multiplication of positive effects and in the joint lifting of potential barriers through effective multilevel governance, especially in the decarbonisation of the transport sector, where responsibilities typically transcend urban boundaries.

## Declaration of competing interest

The authors declare that they have no known competing financial interests or personal relationships that could have appeared to influence the work reported in this paper.

## Data Availability

Data will be made available on request.

## List of abbreviations

CEF: Connecting Europe Facility
CORDIS: Community Research and Development Information Service
FP: Framework Programme
GDP: Gross Domestic Product
GHG: Greenhouse Gas
JPI: Joint Programing Initiative
H2020: Horizon 2020 programme
R&I: Research and Innovation
SET: Strategic Energy Technology
STRIA: Strategic Transport Research and Innovation Agenda
TRIMIS: Transport Research and Innovation Monitoring and Information System
